# Investigation of error detection capabilities of phantom, EPID and MLC log file based IMRT QA methods

**DOI:** 10.1002/acm2.12114

**Published:** 2017-06-06

**Authors:** Dewayne L. Defoor, Sotirios Stathakis, Joseph E. Roring, Neil A. Kirby, Panayiotis Mavroidis, Mohammad Obeidat, Nikos Papanikolaou

**Affiliations:** ^1^ Cancer Therapy and Research Center – Department of Radiation Oncology University of Texas Health Science Center – San Antonio San Antonio TX USA; ^2^ Department of Radiation Oncology University of North Carolina School of Medicine Chapel Hill NC UA

**Keywords:** IMRT, QA Error

## Abstract

A patient specific quality assurance (QA) should detect errors that originate anywhere in the treatment planning process. However, the increasing complexity of treatment plans has increased the need for improvements in the accuracy of the patient specific pretreatment verification process. This has led to the utilization of higher resolution QA methods such as the electronic portal imaging device (EPID) as well as MLC log files and it is important to know the types of errors that can be detected with these methods. In this study, we will compare the ability of three QA methods (Delta^4^®, MU‐EPID, Dynalog QA) to detect specific errors. Multileaf collimator (MLC) errors, gantry angle, and dose errors were introduced into five volumetric modulated arc therapy (VMAT) plans for a total of 30 plans containing errors. The original plans (without errors) were measured five times with each method to set a threshold for detectability using two standard deviations from the mean and receiver operating characteristic (ROC) derived limits. Gamma passing percentages as well as percentage error of planning target volume (PTV) were used for passing determination. When applying the standard 95% pass rate at 3%/3 mm gamma analysis errors were detected at a rate of 47, 70, and 27% for the Delta^4^, MU‐EPID and Dynalog QA respectively. When using thresholds set at 2 standard deviations from our base line measurements errors were detected at a rate of 60, 30, and 47% for the Delta^4^, MU‐EPID and Dynalog QA respectively. When using ROC derived thresholds errors were detected at a rate of 60, 27, and 47% for the Delta^4^, MU‐EPID and Dynalog QA respectively. When using dose to the PTV and the Dynalog method 11 of the 15 small MLC errors were detected while none were caught using gamma analysis. A combination of the EPID and Dynalog QA methods (scaling Dynalog doses using EPID images) matches the detection capabilities of the Delta^4^ by adding additional comparison metrics. These additional metrics are vital in relating the QA measurement to the dose received by the patient which is ultimately what is being confirmed.

## 1 INTRODUCTION

Pretreatment quality assurance (QA) of an intensity modulated radiotherapy (IMRT) plan is essential in preventing errors from propagating throughout the course of treatment. The rate and frequency of potential errors have increased as treatments shifted from 3D conformal to IMRT and volumetric modulated arc therapy (VMAT). Numerous studies have shown the need for QA methods to be appropriate for the treatment technology used.^1^ Therefore, awareness of the error detection capabilities of an IMRT QA system used in clinical environment is important for optimum delivery of complex treatments.

Two‐dimensional (2D) diode arrays are commonly used for patient‐specific IMRT QA. Dose distributions are first calculated on the phantom geometry in the treatment planning system (TPS) and then delivered and measured at the treatment machine. A very common method to quantitatively compare measured and calculated dose distributions is the concept of gamma index introduced by Low et al.^2^ This approach represents the minimum distance between the measurement and calculation points compared against acceptance criteria for distance‐to‐agreement (DTA) and percentage dose differences (%DD). The effectiveness of the gamma index tool for IMRT QA has been investigated^3^, ^4^, ^5^, ^6^, ^7^, ^8^, ^9^ with special emphasis on the sensitivity of different gamma criteria to positioning errors.^5^ The results of these investigations have shown that the gamma index can fail to detect errors that may have a significant biological impact. Fundamentally, the patient‐specific QA should be linked with treatment outcomes.^10^

Dose volume histogram (DVH) based analysis of IMRT QA have proven the effectiveness of this modality while also showing that the gamma index values may have low correlation with the clinical impact of the errors.^4^, ^11^ For this reason, in this study both the gamma index and DVH based metrics will be used to evaluate error detection capabilities of three IMRT patient specific QA methods. Method 1 uses the Delta^4^ (Scandidos, Madison, WI, USA) which is our current clinical IMRT QA system. Method 2 is an in‐house MatLab program (MU‐EPID) (The MathWorks, Inc., Natick, MA, USA) which utilizes the EPID for the purpose of patient specific IMRT QA measurements.^12^ MU‐EPID has previously been validated as an IMRT‐QA tool by comparison with the Delta^4^.^12^, ^13^ Method 3 uses Varian (Varian Medical Systems, Inc, Palo Alto, CA, USA) MLC log files (Dynalogs) and an in‐house MatLab program (Dynalog QA) to import the recorded machine parameters into the treatment planning system (TPS) for patient dose recalculation.

The purpose of this study is to investigate the ability of each method mentioned above to detect intentional errors. It is important for the physicists performing IMRT QA and analyzing the results to know the limitations of the measuring and analysis system. Furthermore, the sensitivity of error detection using two independent methods is examined.

## 2 METHODS AND MATERIALS

### 2.A Patient population

Our patient population consisted of five VMAT patients, four prostate cancer patients, and one rectal cancer patient. These disease sites were chosen because of the similar organs‐at‐risk (OAR) involved in the treatments so direct comparison of DVH values is possible. All VMAT plans were designed using Pinnacle v9.8 (Philips Radiation Oncology Systems, Fitchburg, WI, USA). Each VMAT plan consisted of two full arcs with the isocenter at the center of the planning target volume (PTV). All contours for the target and the OARs were outlined by the same physician. The optimized plans were then copied and calculated on the Delta^4^ phantom using the same monitor units (MU's) as the actual treatment plan. The patient‐specific VMAT QA plans were exported using DICOM to the Scandidos software for comparison against the measurements. Table 1 shows the monitor units (MUs) and beam energies for each of the patient plans.

**Table 1:** Patient parameters

Site	MUs	Energy
Prostate	646	10 MV
Prostate	542	10 MV
Rectum	516	6 MV
Prostate	1322	10 MV
Prostate	648	6 MV

### 2.B Baseline measurements

All plans were optimized and delivered on a Novalis Tx (Varian Medical Systems) linear accelerator equipped with an HD‐120 MLC and an aS1000 EPID. To determine the threshold of detectability of our methods, we first established a baseline by measuring all treatment plans five (*n* = 5) consecutive times with each QA method and performed gamma index analysis of each. Each treatment plan was delivered on the Novalis Tx consecutively on the same day to minimize linac output and EPID response variation. The mean gamma index passing percentage rate for each plan using 3%/3 mm, 2%/2 mm, and 1%/1 mm tolerances was used as a reference standard. Using the baseline statistics, a detection threshold was set at two standard deviations from the mean. The baseline measurements also provide values for D_2_ and D_98_ of the PTV to be used for determination of detection thresholds. Tables 2 and 3 show the resulting gamma index and the average deviation of D_2_ and D_98_.

**Table 2:** Baseline gamma data for all methods and gamma criteria

Delta^4^	SD	Average
3%/3 mm	1.01	98.81%
2%/2 mm	7.42	92.97%
1%/1 mm	15.52	70.76%
Dynalog		
3%/3 mm	0.18	99.86%
2%/2 mm	0.45	99.56%
1%/1 mm	1.31	97.89%
MU‐EPID		
3%/3 mm	1.76	94.03%
2%/2 mm	2.90	82.37%
1%/1 mm	3.03	54.33%

**Table 3:** Baseline dosimetric data for the Dynalog and MU‐EPID methods

Dynalog	SD	Average dev
D_2_	0.37	0.44%
D_98_	0.66	1.67%

MU‐EPID	SD	Average dev
D_2_	1.34	5.20%
D_98_	1.40	9.58%

### 2.C Errors

**Figure 1:**
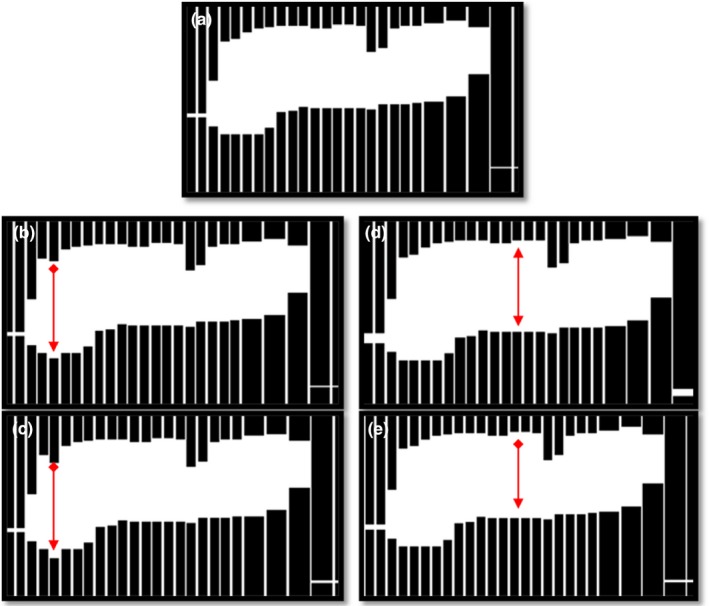
Example of introduced MLC errors. (a) Unmodified plan. (b) One leaf shifted by 2 mm. (c) One leaf shifted by 4 mm. (d) Gap widened by 1 mm. (e) All leaves shifted in the same direction by 1 mm.

A total of six different versions of the original treatment were produced for each patient plan, each version containing a different type of error listed in Table 4. These “deliberate” errors represent typical errors that could occur during treatment. (a) Dose errors were simulated by increasing the overall delivered MU by 3%. (b) Gantry angle errors were introduced by increasing the gantry angle by 1 degree at each control point. (c) A 2 mm MLC shift was introduced to the center leaf pair at each control point. (d) The same leaves were also shifted by 4 mm in a separate plan. (e) A plan was created in which all MLC pairs were shifted by 1 mm at every control point, and (f) another plan in which all MLC leaves were shifted away from the central axis by 1 mm. Such shifts of all MLC leaves were chosen to represent an MLC calibration error. The plans were modified to include the errors above, using a MatLab script and the original DICOM RT file for each patient. Figure 1 shows a visual representation of the MLC errors.

**Table 4:** Errors introduced into VMAT plans

Errors	Magnitude
Dose	3%
Gantry rotation	1 degree
Leaf pair shift	2 mm
Leaf pair shift	4 mm
All leaves shifted	1 mm
MLC banks widened	1 mm

### 2.D Receiver operating characteristic (ROC) analysis

**Figure 2:**
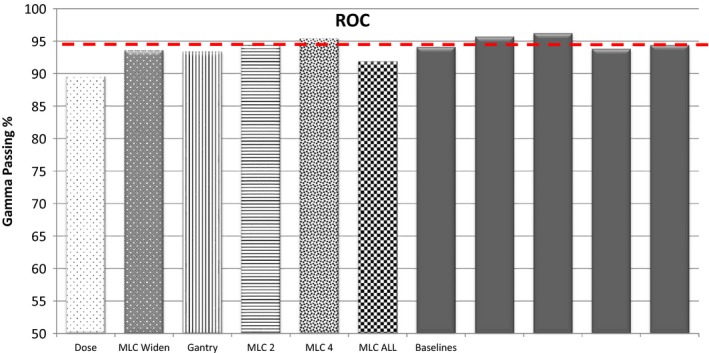
Example of determination of ROC curves.

**Figure 3:**
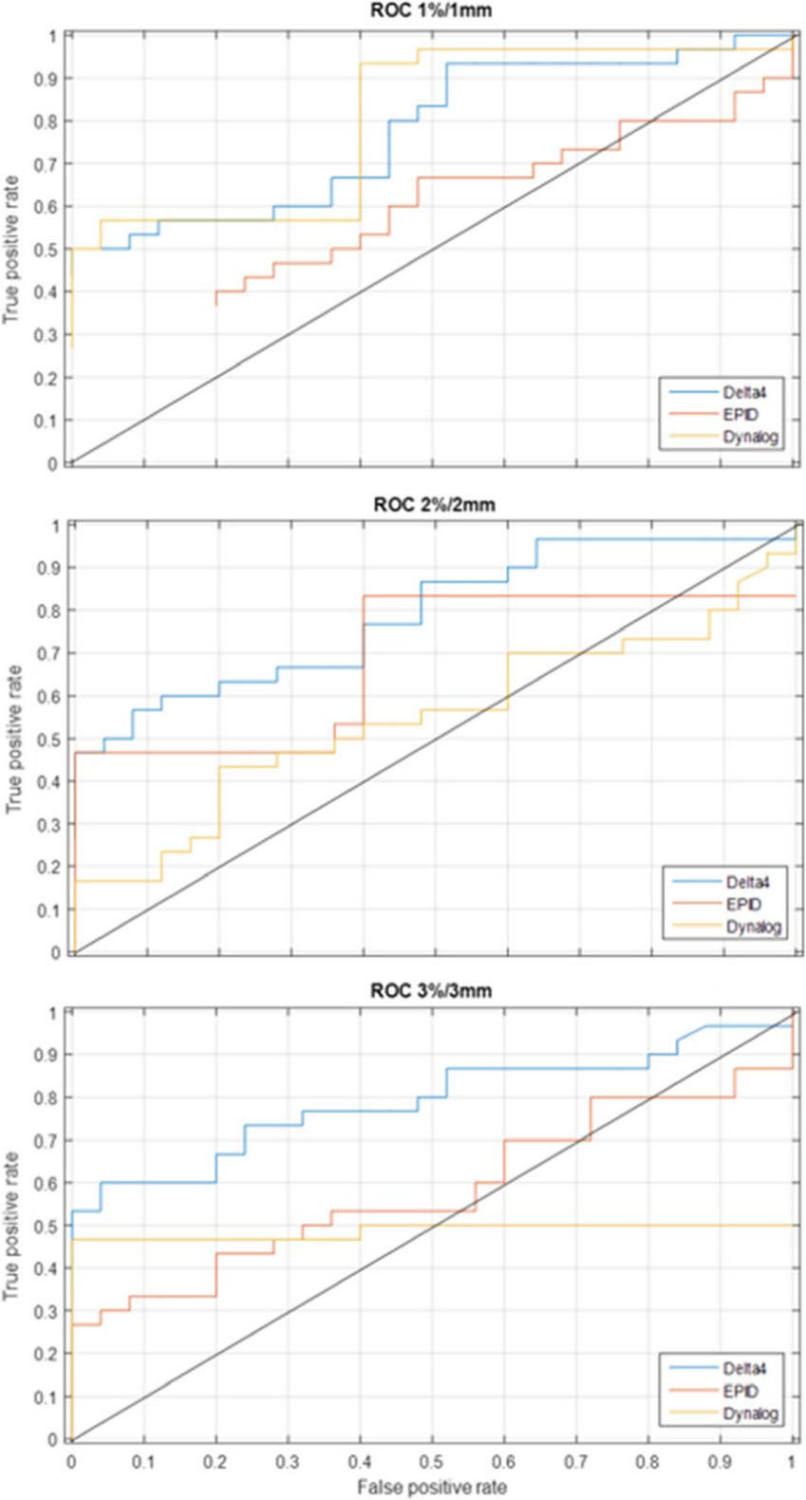
ROC curves for all three methods.

ROC analysis provides tools to identify optimal models and to discard suboptimal ones. ROC analysis is to cost/benefit analysis of diagnostic decision‐making. An ROC curve is generated by quantifying true positives (errors detected) and false positives (detections that are not errors) and plotting their respective probabilities. To investigate the detectability of each method, the number of true positives and false positives were determined for many different thresholds (Fig. 2) for the gamma criteria of 3%/3 mm, 2%/2 mm, and 1%/1 mm. These data were used to generate ROC curves (Fig. 3). For each method, results are presented for the gamma criteria that resulted in the largest area under the ROC curve and the threshold chosen was that which had the highest true positive to false positive ratio. If a threshold had zero false positives the ratio was just the number of true positives. Thresholds for the PTV dose analysis were determined using this method as well.

### 2.E Scandidos delta^4^

The treatment plans containing “known” errors, for each patient, were measured using the Delta^4^ detector array, following our institutional procedures. Gamma analysis of the Delta^4^ measurements was performed with the Delta^4^ software. The phantom position was then optimized using the Delta^4^ software option to correct for phantom set up errors. The gamma index was then calculated for the criteria mentioned previously in the Baseline Measurements section.

### 2.F Epid

The IMRT QA plans were delivered without a phantom in the beam and the fluence maps were collected by the EPID. The EPID images were processed through the MU‐EPID software for all patients to convert them into an optical density matrix (ODM) and imported into Pinnacle TPS. First, a pixel‐intensity‐to‐MU conversion factor was determined by delivering 100 MU using a 10 × 10 cm^2^ field to the EPID prior to each IMRT QA session. An average pixel intensity is taken over a region of the calibration image corresponding to the central axis of the beam. This pixel value per MU factor was then used to convert IMRT QA image intensities to MU values that were applied to the ODMs. The IMRT QA images were then corrected for the variation in response across the EPID by applying a correction matrix. The correction matrix was created by comparing dose resulting from an ideal fluence to dose resulting from the EPID detected fluence. Using the ODMs, a plan was calculated in Pinnacle using the same patient geometry and contours. The DVHs for each ODM based plan were then calculated using the treatment planning system. The doses calculated using each field's ODM in Pinnacle for each patient plan were then exported to the MU EPID software. Dose distributions and DVHs for the initial plans and the recalculated ones (using the EPID based ODM) were compared for each plan.

### 2.G Dynalog

The Dynalog files were recorded during the EPID image acquisitions. The MLC positions, gantry angles and collimator angles were collected and used to replace the respective ones of the original plans and imported back to Pinnacle TPS. The treatment plans were then recalculated using the dynalog recorded parameters for each patient. The resulting dose distributions were exported and processed using MatLab to obtain the same dosimetric data as with the plans calculated using ODMs generated from EPID images. Dynalog files do not contain information on the actual number of monitor units delivered, therefore EPID images were used in conjunction with MU values derived from the same pixel‐intensity‐to‐MU conversion algorithm used in the EPID method.

### 2.H Gamma index analysis

Gamma index analysis was performed in Verisoft (PTW, Freiburg, Germany) for the MU‐EPID and Dynalog QA methods while the Scandidos Delta^4^ software was used for the Delta^4^ measurements. A dose grid resolution of 0.3 cm was used for MU‐EPID and Dynalog QA dose distributions and 3D gamma analysis was performed. The 2D gamma analysis used in the Delta^4^ software compares dose measured at each diode (0.5 cm resolution in the center 6 cm × 6 cm area and 1 cm resolution elsewhere) with the dose calculated by the TPS. A total of 55 treatment plans were analyzed with each method, 30 error containing plans and 25 plans without errors to establish a baseline. The detection of errors was evaluated at the standard passing gamma percentages 90% at 3%/3 mm, 80% at 2%/2 mm, and 50% at 1%/1 mm. The passing criteria for 2%/2 mm and 1%/1 mm were derived by matching the number of detected errors at the standard 90% passing threshold. Error detection was also evaluated at passing thresholds set at two standard deviations (SD) from the mean of the baseline and at the percentage determined from ROC analysis.

## 3 RESULTS

### 3.A Gamma index analysis

**Figure 4:**
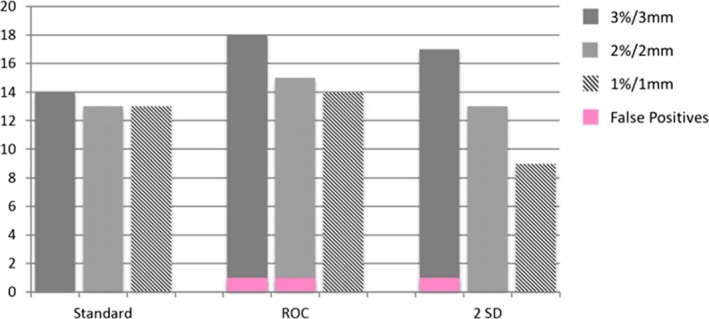
Bar graphs of errors detected by the Delta^4^ at 3%/3 mm, 2%2/ mm, and 1%1 mm.

**Figure 5:**
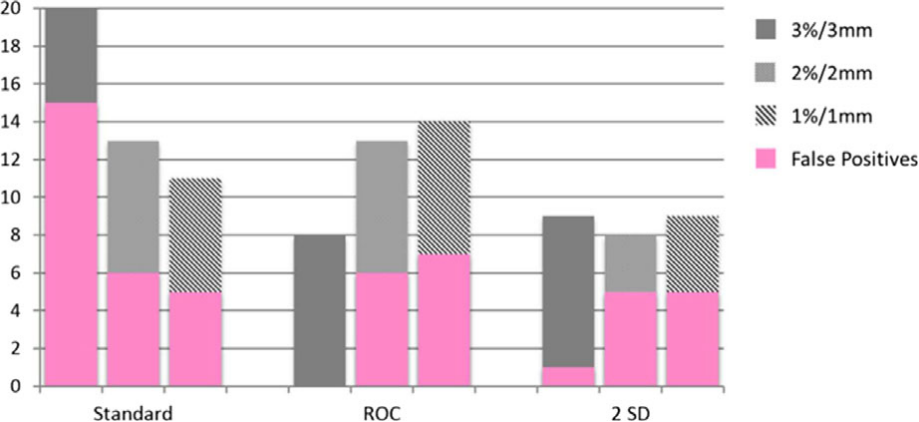
Bar graphs of errors detected by MU‐EPID at 3%/3 mm, 2%2/ mm, and 1%1 mm.

**Figure 6:**
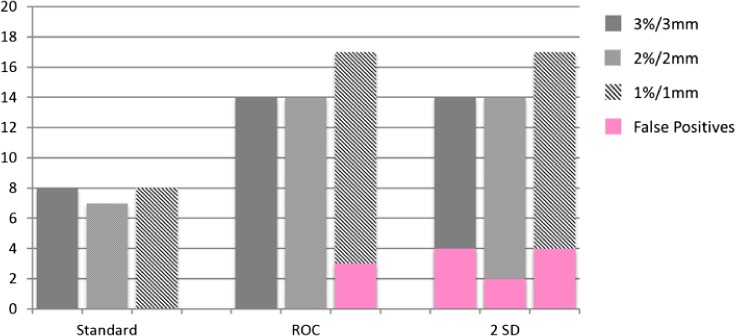
Bar graphs of errors detected by Dynalog QA at 3%/3 mm, 2%2/ mm, and 1%1 mm.

At 3%/3 mm and the ROC derived passing gamma cut‐off, the Delta^4^ (96.9%) detected 18 of 30 errors with 1 false positive, the EPID (90%) detected 8 of 30 errors with 0 false positives and the MLC log file method (99.4%) detected 14 of 30 errors with 0 false positives. At 3%/3 mm and the 2 SD passing gamma cut‐off, the Delta^4^ (96.8%) detected 18 of 30 errors with one false positive, the EPID (90.5%) detected 9 of 30 errors with one false positive and the MLC log file method (99.5%) detected 14 of 30 errors with four false positives. Evaluation of gamma at 3%/3 mm yielded better ROC curves than 2%/2 mm or 1%/1 mm. This is due to the increase in the number of false positives with the more strict criteria. Bar graphs showing the number of errors detected along with the false positives (pink) using gamma analysis are shown in Figs. 4, 5, 6.

### 3.B PTV dose

**Figure 7:**
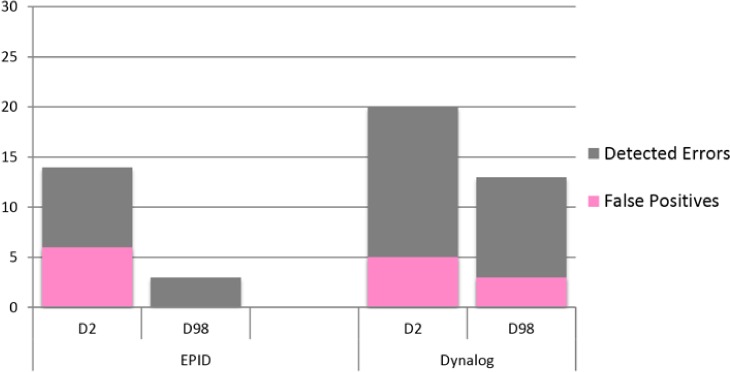
Bar graphs of errors detected by using D2 and D98 of the PTV.

The dose comparison metrics between the plans containing errors and the original plans were the absolute percent difference of D_2_ and D_98_ of the PTV. The threshold for detection of errors based on D_2_ and D_98_ was determined by ROC analysis. Using D_2_ of the PTV and a detection threshold of 0.8% the Dynalog method detected 20 of 30 errors with five false positives. However, false positives were detected for only one patient with all five baseline measurements having PTV D_2_ deviations of greater than 1.4%. The maximum PTV D_2_ deviation for all other baseline measurements of the other four patients was 0.3%. Using a detection threshold of 6%, the EPID method detected 14 of 30 errors with six false positives. Finally, using D_98_ of the PTV and a detection threshold of 5.2% the Dynalog method detected 13 of 30 errors with three false positives, and with a detection threshold of 16% the EPID method detected 3 of 30 errors with zero false positives. A bar graph depicting the errors detected and the number of false positives using D_2_ and D_98_ is shown in Fig. 7.

## 4 DISCUSSION

### 4.A Gamma analysis

#### 4.A.1 MU‐EPID

The EPID showed limited error detection capabilities mainly due to the geometry (loss of gantry angle information) and the over response to scattered radiation. Corrections for the variation in response across the EPID were applied, however, the correction is not able to account for scatter radiation which will largely depend on the plan being delivered. These deficiencies limit the effectiveness of the EPID as a comprehensive IMRT QA tool. The EPID does, however, provide a physical “measurement” that can be useful. The pixel intensity to MU conversion factor derived from the 100 MU calibration image has shown a variation of less than 1%. The resulting MU value can be used in conjunction with log file based IMRT QA methods to incorporate a “measurement” into the process. This method was used with the Dynalog QA method investigated here.

### 4.B Dynalog QA

The Dynalog method performed similarly to the Delta^4^ when using the 2 SD and ROC derived pass thresholds. Dynalog method did not have trouble detecting the gantry angle errors but had difficulty detecting small MLC deviations.

### 4.C Delta^4^

The Delta^4^ was deficient in detecting gantry angle errors. The Delta^4^ uses gantry angles supplied in the treatment plan and not the actual gantry angles and therefore does not recognize gantry angle deviations. The Delta^4^ was also unsuccessful in detecting small MLC deviations. This is due to the relatively large diode spacing compared to the magnitude of the MLC errors.

### 4.D Dose to PTV

The Dynalog method performed very well when using the dosimetric indicators. Where gamma analysis had failed to detect small MLC deviations, D_2_ was successful. With this detection method 11 of the 15 small MLC errors were detected while a total of seven were caught using gamma analysis: 6 of 15 with the Delta^4^, 1 of 15 with the EPID and none with Dynalogs. The EPID method performed poorly here because of the blurring of the dose distribution due to scatter radiation. There were large differences seen in both the original plans as well as with the error containing plans with the EPID method which made it difficult to attribute deviations to errors.

### 4.E Analysis metrics

Gamma index analysis exhibited poor sensitivity to small errors for all three IMRT QA methods investigated here. The failure of the gamma index to detect small errors has been documented and is in line with our findings.^4^, ^6^, ^8^, ^11^ Even with optimization using ROC methods gamma analysis fails to detect MLC errors of less than ~2 mm.^14^ However, the use of D_2_ as an additional error detection metric enhances the capabilities of the systems to detect small errors as well as reducing the number of false positives that can be an issue with gamma index analysis.

## 5 CONCLUSION

Measurements to established baseline data showed significant difference in the average gamma values between methods especially for stricter gamma index calculation criteria. The baseline data were used to perform ROC analysis to determine the error detection capabilities of each patient specific QA method. Our results from all patient measurements highlighted the strengths and weaknesses of each method used for patient specific QA in detecting small clinically relevant errors. It was also evident from the results that the gamma index as an analysis tool has significant limitations in detecting small errors as no method investigated here could detect more than 60% of the intentional errors.

Individually, neither the EPID nor Dynalog method performed as well as the Delta^4^ when using the gamma index only. However, a combination of the two methods (scaling Dynalog doses using EPID images) matches the detection capabilities of the Delta^4^. Moreover, with the scaled Dynaolgs, the plan can be recalculated using the MLC location information and using the PTV D_2_ and D_98_, we were able to increase the detectability of the errors. The increased sensitivity gained for analysis of dose to the PTV combined with phantom‐less data collection makes this method an attractive alternative to phantom‐based patient specific IMRT QA.

## CONFLICT OF INTEREST

We have no conflict of interest to declare.
